# Determinants of comprehensive HIV and AIDS knowledge among Tanzanian adolescent girls and young women: a multilevel application of Andersen’s behavioral model based on national survey

**DOI:** 10.3389/fpubh.2025.1643422

**Published:** 2025-09-09

**Authors:** Mussa Hassan Bago, Elihuruma Eliufoo Stephano, Sahnun Ally Kessy, Jovin R. Tibenderana, Victoria Godfrey Majengo, Erick Donald Oguma, Tegemea Patrick Mwalingo, Immaculata P. Kessy, Azan Abubakar Nyundo, Mtoro J. Mtoro

**Affiliations:** ^1^Department of Public Health and Community Nursing, School of Nursing and Public Health, The University of Dodoma, Iyumbu, Tanzania; ^2^Department of Clinical Nursing, School of Nursing and Public Health, The University of Dodoma, Iyumbu, Tanzania; ^3^Directorate of Research and Training, Benjamin Mkapa Hospital, Iyumbu, Tanzania; ^4^Department of Public Health, St. Francis University, College of Health and Allied Sciences, Ifakara, Tanzania; ^5^Department of Obstetrics and Gynecology, Dodoma Regional Referral Hospita, lDodoma, Tanzania; ^6^TILAM International, Dar es Salaam, Tanzania; ^7^Department of Psychiatry and Mental Health, School of Medicine and Dentistry, The University of Dodoma, Iyumbu, Tanzania

**Keywords:** knowledge, adolescent girls, young women, HIV, aids

## Abstract

**Background:**

Limited comprehensive HIV and AIDS knowledge is a significant factor contributing to the prevalence of HIV among adolescent girls and young women (AGYW). Therefore, this study aimed to determine the level of comprehensive HIV and AIDS knowledge and identify its associated factors among AGYW in Tanzania.

**Methods:**

An Analytical cross-sectional study of the 2022 Tanzania Demographic and Health Surveys data was conducted. The study included 5,810 AGYW, selected through a two-stage sampling method. Multilevel logistic regression, accounting for the complex survey design, was used to identify individual and community-level factors associated with comprehensive HIV/AIDS knowledge. Analyses were conducted using Stata 18.5. Adjusted odds ratios (AORs) with 95% confidence intervals (CIs) were reported, and statistical significance was set at *p* < 0.05.

**Results:**

The prevalence of comprehensive HIV and AIDS knowledge among AGYW was 46.2% (95% CI: 44.3–48.2%). In the final fitted multivariable analyses, AGYW in primary (AOR = 1.97, 95%CI: 1.56–2.47), secondary (AOR = 3.79, 95%CI: 2.96–4.85), AGYW in a rich quantile (AOR = 1.38, 95%CI: 1.15–1.66), owning a mobile phone (AOR = 1.26, 95%CI: 1.09–1.45), the use of the internet (AOR = 1.40, 95%CI: 1.16–1.68) and AGWY who ever tested for HIV (AOR = 1.61, 95%CI: 1.39–1.87) had higher odds of having comprehensive knowledge compared to their counterparts. At the community level, geographical zones exhibited higher odds of having comprehensive knowledge.

**Conclusion:**

Despite notable progress in HIV education, gaps persist, especially among AGYW, underscoring the need for focused, diversified interventions. By advancing understanding of these determinants, the study provides crucial evidence to inform tailored HIV prevention strategies aimed at enhancing knowledge and reducing infection rates within this vulnerable population and improving safer sex practices.

## Background

Globally, Human Immunodeficiency Virus and Acquired Immune Deficiency Syndrome (HIV and AIDS) remain a deadly pandemic and a pressing public health concern, with sub-Saharan Africa (SSA) bearing a significant burden (1–3). Over 38 million people are infected with HIV, with women accounting for more than half (19.2 million) of the infected population. Of these, 15.9 million are women in SSA (3–5). Despite representing a small fraction of the population, young women and teenage girls aged 15 to 24 years comprise 25% of new infections in SSA, and are nearly three times more likely to acquire an HIV infection than their male peers (3, 4). Every week, approximately 3,100 adolescent girls and young women (AGYW) in SSA become infected with HIV (5). AIDS is currently the leading cause of death among adolescents in Africa and the second leading cause of adolescent deaths worldwide, with SSA having the highest number of AIDS-related adolescent deaths (6). Tanzania remains one of the high HIV burden countries in Africa. Although the HIV prevalence among individuals aged 15–49 years has progressively declined from 7.0% in 2003/2004 to 5.7% in 2007/2008, 5.1% in 2011/2012, and 4.7% in 2016/2017, approximately 1.7 million people are currently living with HIV (PLHIV) (7, 8). Notably, HIV prevalence in the country varies widely by region, ranging from 0.3 to 11.6%, with the southern highlands reporting the highest rates (8, 9). Meanwhile in Zanzibar-a semi-autonomous region of the United Republic of Tanzania has an overall HIV prevalence of below 1% in the general population (8). This contrast with mainland Tanzania may be partly attributed to Zanzibar’s proactive health education policies. For example, the Zanzibar Ministry of Education identified primary school as a critical entry point for HIV and AIDS prevention education, aiming to reach children before sexual debut (10).

In 2021, 5.1% of Tanzania’s population was reported to be HIV-infected, while 2% of youth aged 15–24 years are HIV-positive (11). The early sexual initiation of adolescents in Tanzania has been reported to be less than 15 years; hence, the higher risk of acquiring HIV/AIDS and other sexually transmitted diseases (12). Furthermore, evidence suggests that high-risk sexual behaviors, low knowledge of one’s HIV serostatus, low level of knowledge and awareness about HIV and AIDS (13), socioeconomic disparities, health inequalities, gender inequality, abuse of power, cultural supremacies and stigmatization of people living with HIV have barred comprehensive and coordinated global action to prevent HIV infections among young women (4, 14–16). To reduce the risk of HIV infection and transmission, it’s important to consider various contributing factors. However, many of the factors are often difficult to modify, such as a country’s health infrastructure, demographics, economy, and political climate (17). Therefore, focusing on increasing public comprehensive knowledge about HIV and AIDS is a more hands-on and impactful approach.

Though women of reproductive age in SSA share the greatest burden of the HIV and AIDS epidemic and are at higher risk of acquiring the infection (2), comprehensive knowledge about HIV and AIDS among this group in the region was found to be as low as 38.56% according to a multilevel analysis of DHS data conducted in 2022 (18). Various studies conducted across Africa have shown that fewer than half of women of reproductive age have demonstrated a low comprehensive knowledge of HIV/AIDS. For example, in Ethiopia, only 25.2% of women aged 15–49 years (14). Another study was done in three East African countries; Burundi, Kenya and Ethiopia among women living with HIV/AIDS aged (15–49) also showed a comprehensive knowledge on HIV/AIDS being less than half: Burundi (48.9%), Kenya (46.3%), while in Ethiopia was below one-fifth (19.3%) (19).

Currently, to the best of our knowledge, there has been no published study on comprehensive knowledge about HIV and AIDS and their associated factors among AGYW in Tanzania. Hence, this study assesses the level of comprehensive knowledge about HIV and AIDS and its associated factors among AGYW in Tanzania using a nationally representative dataset. The evidence-based insights generated from this study will help stakeholders, policymakers, and program planners in guiding programmatic efforts and the strengthening of existing health education frameworks that could potentially increase HIV and AIDS awareness in order to end the pandemic.

## Methods and materials

### Conceptual framework

**Figure 1:**
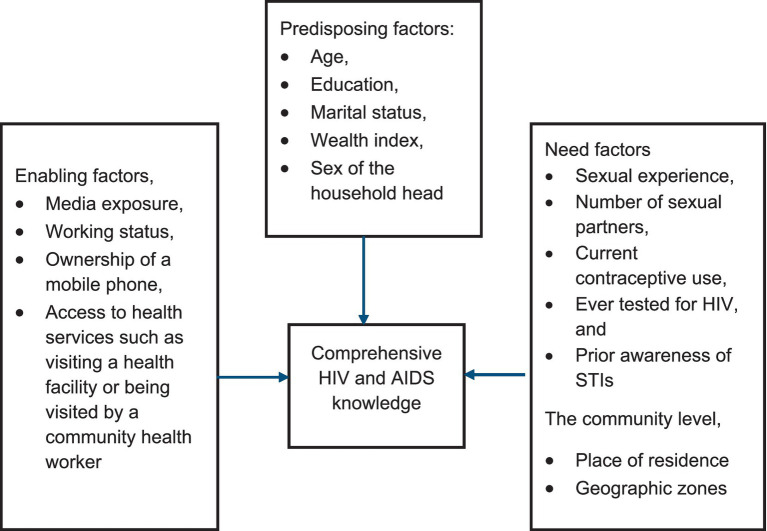
Conceptual framework for assessing the determinants of comprehensive HIV and AIDS knowledge.

This study utilizes the guide of Andersen’s Behavioral Model, which helped us to structures the investigation by showing how demographic predispositions, enabling resources, and health needs at both individual and community strata collectively determine comprehensive HIV and AIDS knowledge outcomes (20). The application of this model provides a comprehensive multilevel conceptual framework to guide the study on correlates of comprehensive HIV and AIDS knowledge among adolescent girls and young women in Tanzania by systematically organizing individual and community-level determinants influencing health knowledge acquisition. The model was previously utilized using the Tanzania Demographic and Health Surveys (TDHS) in assessing HIV testing (21). At the individual level, predisposing factors such as age, education, marital status, wealth index, and sex of the household head reflect intrinsic demographic characteristics and socioeconomic status that shape adolescents’ propensity to acquire HIV and AIDS knowledge. Enabling factors, including media exposure, working status, ownership of a mobile phone, and access to health services such as visiting a health facility or being visited by a community health worker, represent resources that facilitate information access and health knowledge dissemination. Additionally, need factors such as sexual experience, number of sexual partners, current contraceptive use, ever tested for HIV, and prior awareness of STIs signify perceived health-related requirements motivating the pursuit of HIV and AIDS information. At the community level, contextual enabling factors like place of residence and geographic zones capture environmental influences shaping access to and availability of HIV and AIDS educational resources. The dependent variable, comprehensive HIV and AIDS knowledge, operationalized as correct understanding across critical misconceptions and prevention knowledge, is influenced by the interplay of these multilevel factors. See Figure 1.

### Data source and design

This study was an analytical cross-sectional survey that utilized secondary data from the 2022 TDHS, which conducts nationally representative population-based household surveys typically every five years, the survey which involve the Tanzania National Bureau of Statistics and the Ministries of Tanzania Mainland and Zanzibar.

### Population and sampling

Data for this study were obtained from the latest DHS conducted between 24 February and 21 July 2022 across all regions in Tanzania. The target population for the 2022 TDHS included women of reproductive age (15–49 years) across the 32 administrative regions in Tanzania. At the country level, a sampling frame is usually obtained. To minimize sampling errors, the country is stratified by geographic region and by urban/rural areas within each region, followed by a two-stage sampling to select a household to be surveyed. The first sampling is to select a primary sampling unit (PSU) and then select a household. PSUs are survey clusters that are usually based on census enumeration areas (EAs). A probability proportion to size is employed in each stratum to select the PSU. For each selected PSU, a complete household listing is done. This is then followed by selecting a fixed number of households to be surveyed using equal probability systematic sampling. All women who had spent the night before the survey in the selected households were eligible for the survey. We began with 15,254 records from the individual file (IR) of participants aged 15–49 years. For this analysis, which focused specifically on AGYW aged 15–24 years, we excluded individuals aged 25 and older, resulting in a final sample size of 5,810 (weighted) participants.

### Study variables

#### Dependent variable

The outcome variable of the study was comprehensive knowledge about HIV and AIDS, defined as a composite measure based on responses to five yes/no questions: (1) Consistent condom use during sex reduces the risk of HIV infection; (2) Having a single, uninfected, and monogamous partner reduces the risk of HIV; (3) A healthy-looking person can be HIV-positive; (4) HIV can be transmitted through mosquito bites; and (5) HIV can be transmitted by sharing food with someone who has AIDS. Adolescents were considered to have comprehensive knowledge if they answered all five questions correctly, responding “Yes” to questions 1, 2, and 3, and “No” to questions 4 and 5.

#### Independent variables

The current study examined variables at the individual and community levels based on the available data and literature. The following variables were included under the individual level; age in years (15–19 or 20–24), education level (no formal education, primary, secondary or higher), marital status (never married, married), wealth index (poor, middle or riche), media exposures (yes as listening to radio, reading newspaper or watching Television less than once a week or at least once a week or no if otherwise), ever had sex (yes or no), working status (working or not working), number of sexual partners (none, one or more), current contraceptive use (none or any method), sex of household head (male or female), owns mobile phone (yes or no), visited health facility in the past 12 months (yes or no), visited by community health worker (yes or no), ever tested for HIV (yes or no) and ever heard of STI (yes or no). The following variables were included under the community level: place of residence (urban or rural) and geographical zones (western, northern, central, southern, eastern or Zanzibar).

#### Data management and analysis

To address the complex survey design of the TDHS, we applied individual sampling weights (v005/1,000,000), accounted for primary sampling units (clusters), and stratified the data to ensure representative estimates and control for sampling biases. Data cleaning, coding, and analysis were performed using STATA 18.5 (STATA Corp, College Station, TX). Descriptive statistics included means and standard deviations for continuous variables, and frequencies with proportions for categorical variables. Considering the hierarchical structure of the data (women within households within clusters), we employed a multilevel mixed-effects logistic regression model to account for non-independence and unequal variance. Our analysis involved four models: a null model (Model 0), a model with individual-level factors (Model I), a model with community-level factors (Model II), and a final model combining both levels (Model III).

### Random effects and model fitness

To assess the variation in comprehensive HIV/AIDS Knowledge across clusters, we calculated random effects measures including the Intra-class Correlation Coefficient (ICC), Median Odds Ratio (MOR), and Proportion Change in Variance (PCV). Treating clusters as a random effect, the ICC, quantifying the proportion of total variation in comprehensive HIV/AIDS Knowledge use attributable to between-cluster variations, was computed as ICC = [VC / (VC + 3.29)] * 100. The MOR, representing the median odds ratio between clusters with high and low likelihood of comprehensive HIV/AIDS knowledge, was calculated as MOR = exp. [0.95 * sqrt (VC)]. The PCV, indicating the reduction in variance across successive models, was calculated as (Ve – Vmi)/Ve, where Ve is the variance in the null model and Vmi is the variance in subsequent models.

The association between individual and community-level independent variables and the likelihood of comprehensive HIV/AIDS Knowledge was estimated using fixed effects, presented as adjusted odds ratios (AORs) with 95% confidence intervals. Model comparison, due to the nested structure, was based on the deviance statistic (−2 * log-likelihood), with lower deviance and higher log-likelihood indicating a better fit. Multilevel logistic regression analysis was performed using the ‘melogit’ package in Stata. Prior to multivariable regression modelling, multicollinearity among independent variables was assessed using the Variance Inflation Factor (VIF). All analyses were two-sided, and statistical significance was set at *p* < 0.05.

## Results

### Sociodemographic characteristics and prevalence of comprehensive knowledge

The prevalence of comprehensive HIV and AIDS knowledge among AGYW was 46.2% (95% CI: 44.3–48.2%). Knowledge levels significantly varied by age, education, wealth status, media exposure, household size, working status, mobile phone ownership, internet use, health service utilization, and residence (*p* < 0.05). Higher knowledge was observed among respondents aged 20–24 years (49.5%), those with secondary or higher education (57.1%), from wealthier households (53.7%), and those with media (50.7%) or internet exposure (62.8%). Urban residents (54.3%) and those who had visited a health facility in the past 12 months (49.8%) or had been tested for HIV (50.8%) also demonstrated higher knowledge levels. In contrast, marital status, sex of the household head, being visited by a health care worker, and sexual activity history were not significantly associated with comprehensive HIV knowledge (*p* ≥ 0.05) (Table 1).

**Table 1:** Individual and community level characteristics and distribution of comprehensive knowledge of HIV and AIDS in Tanzania among adolescent girls and young women.

Variables	*N* (%)	Comprehensive HIV knowledge, *n* (%)		*p*-value
Prevalence of comprehensive HIV and AIDS knowledge	46.2 (95%CI: 44.3–48.2)	No (*n* = 3,124)	Yes (*n* = 2,686)	
Age in years				<0.001
15–19	3,083 (53.1)	1747 (56.7)	1,336 (43.3)	
20–24	2,727 (46.9)	1,377 (50.5)	1,350 (49.5)	
Marital status				0.150
Never married	3,332 (57.3)	1752 (52.6)	1,580 (47.4)	
Married	2,478 (42.7)	1,372 (55.4)	1,106 (44.6)	
Education level				<0.001
No formal education	636 (10.9)	501 (78.8)	135 (21.2)	
Primary	2,550 (43.9)	1,498 (58.7)	1,052 (41.3)	
Secondary/higher	2,624 (45.2)	1,126 (42.9)	1,498 (57.1)	
Wealth index				<0.001
Poor	1920 (33)	1,260 (65.6)	670 (34.4)	
Middle	1,109 (19.1)	577 (52.1)	532 (47.9)	
Rich	2,782 (47.9)	1,287 (46.3)	1,495 (53.7)	
Media exposure				<0.001
No	1,679 (28.9)	1,088 (64.8)	591 (35.2)	
Yes	4,131 (71.1)	2036 (49.3)	2095 (50.7)	
Household size				<0.001
≤6	3,558 (61.2)	1815 (51.0)	1743 (49.0)	
≥7	2,252 (38.8)	1,309 (58.1)	943 (41.9)	
Sex of household head				0.273
Male	4,209 (72.4)	2,286 (54.3)	1923 (45.7)	
Female	1,601 (27.6)	838 (52.3)	763 (47.7)	
Working status				0.039
Not working	3,089 (53.2)	1713 (55.5)	1,376 (44.5)	
Working	2,721 (46.8)	1,411 (51.9)	1,310 (48.1)	
Owns a mobile phone				<0.001
No	3,290 (56.6)	1976 (60.1)	1,314 (39.9)	
Yes	2,520 (43.4)	1,148 (45.6)	1,372 (54.4)	
Internet use				<0.001
No	5,061 (87.1)	2,846 (56.2)	2,215 (43.8)	
Yes	749 (12.9)	278 (37.2)	471 (62.8)	
Visited health facility past 12 months				0.001
No	3,212 (55.3)	1819 (56.6)	1,393 (43.4)	
Yes	2,598 (44.7)	1,305 (50.2)	1,293 (49.8)	
Visited by CHW in the past 12 months				0.867
No	5,655 (97.3)	3,040 (53.7)	2,615 (46.3)	
Yes	155 (2.7)	84 (54.6)	71 (45.4)	
Current contraceptive use				0.001
No method	4,694 (80.8)	2,603 (55.5)	2091 (44.5)	
Any method	1,116 (19.2)	521 (46.7)	595 (53.3)	
Ever had sex				0.206
No	2085 (35.9)	1,149 (55.1)	936 (44.9)	
Yes	3,725 (64.1)	1975 (53.0)	1750 (47.0)	
Ever heard STI				<0.001
No	1,635 (28.1)	1,228 (75.2)	407 (24.8)	
Yes	4,175 (71.9)	1895 (45.4)	2,280 (54.6)	
Ever tested for HIV				<0.001
No	2,313 (39.8)	1,404 (60.7)	909 (39.3)	
Yes	3,497 (60.2)	1720 (49.2)	1777 (50.8)	
Residence				<0.001
Urban	2075 (35.7)	947 (45.7)	1,128 (54.3)	
Rural	3,735 (64.3)	2,177 (58.3)	1,558 (41.7)	
Geographical zones				0.0123
Western	538 (9.3)	318 (59.1)	220 (40.9)	
Northern	618 (10.6)	379 (61.3)	239 (38.7)	
Central	640 (11.0)	329 (51.4)	311 (48.6)	
Southern	1,077 (18.5)	562 (52.2)	515 (47.8)	
Lake	1746 (30.0)	907 (52.0)	839 (48.0)	
Eastern	980 (16.9)	498 (50.8)	482 (49.2)	
Zanzibar	211 (3.6)	131 (62.1)	80 (37.9)	

CHW, community healthcare worker; STI, sexually transmitted infections.

### Factors associated with comprehensive HIV and AIDS knowledge

In the final fitted multivariable analyses, AGYW in primary (AOR = 1.97, 95%CI: 1.56–2.47) and secondary (AOR = 3.79, 95%CI: 2.96–4.85) had a higher likelihood of having comprehensive knowledge compared to those with no formal education. AGYW in a rich quantile (AOR = 1.38, 95%CI: 1.15–1.66) were 38% more likely to have comprehensive knowledge compared to those in the poor quintile. Owning a mobile phone showed the increased likelihood of having comprehensive HIV/AIDS knowledge (AOR = 1.26, 95%CI: 1.09–1.45), same as the use of the internet (AOR = 1.40, 95%CI: 1.16–1.68). AGWY whoever tested for HIV had higher odds of having comprehensive knowledge compared to their counterparts (AOR = 1.61, 95%CI: 1.39–1.87). At the community level, geographical zones exhibited higher odds of having comprehensive knowledge (Table 2).

**Table 2:** Individual and community level variables associated with basic comprehensive knowledge.

Variables	Model 0	Model I	Model II	Model III
		AOR (95%CI)	AOR (95%CI)	AOR (95%CI)
Individual-level variables				
Age in years				
15–19		1.00		1.00
20–24		1.04 (0.90–1.20)		1.05 (0.91–1.22)
Marital status				
Never married		1.11 (0.95–1.30)		1.11 (0.95–1.30)
Ever married		1.00		1.00
Education level				
No formal education		1.00		1.00
Primary		1.99 (1.58–2.51)**		1.97 (1.56–2.47)**
Secondary/higher		3.47 (2.71–4.45)**		3.79 (2.96–4.85)**
Wealth index				
Poor		1.00		1.00
Middle		1.18 (0.99–1.40)		1.25 (1.04–1.48)*
Rich		1.26 (1.07–1.49)*		1.38 (1.15–1.66)**
Household size				
≤6		1.00		1.00
≥7		0.85 (0.76–0.96)*		0.89 (0.79–1.00)
Has mobile phone				
No		1.00		1.00
Yes		1.26 (1.10–1.45)*		1.26 (1.09–1.45)*
Internet use				
No		1.00		1.00
Yes		1.33 (1.11–1.60)*		1.40 (1.16–1.68)**
Ever tested for HIV				
No		1.00		1.00
Yes		1.67 (1.44–1.94)**		1.61 (1.39–1.87)**
Community-level variables				
Residence				
Urban			1.67 (1.43–1.94)**	1.06 (0.89–1.25)
Rural			1.00	1.00
Geographical zones				
Western			1.30 (0.95–1.77)	2.48 (1.81–3.38)**
Northern			1.12 (0.84–1.49)	1.49 (1.12–1.97)*
Central			1.42 (1.06–1.90)*	2.31 (1.73–3.08)**
Southern			1.61 (1.29–2.02)**	2.39 (1.90–3.00)**
Lake			1.38 (1.10–1.74)*	2.33 (1.84–2.94)**
Eastern			1.40 (1.06–1.83)*	1.89 (1.45–2.48)**
Zanzibar			1.00	1.00
Random effects				
Variance	0.40	0.32	0.30	0.22
PCV	-	20.0%	25.0%	45.0%
ICC	10.9%	8.8%	8.4%	6.1%
MOR	1.12	1.11	1.10	1.09
Model fitness				
AIC	7908.19	7565.22	7857.86	7504.76
BIC	7921.53	7645.31	7917.92	7631.58
Deviance	7904.19	7541.22	7839.86	7466.76

**p < 0.001; *p < 0.05, AIC, Akaike information criterion; BIC, Bayesian information criterion; ICC, intra-cluster correlation coefficient; MOR, median odds ratio; PCV, proportional change in variance.

### Random effects and model fitness

A null model was used to assess whether the data supported the decision to evaluate randomness at the community level. The results from the null model, which showed a variance of 0.40 and a *p*-value of < 0.01, revealed significant variations in comprehensive knowledge across clusters. The variance within clusters contributed 10.9% of the variation in comprehensive knowledge, while the variance across clusters was responsible for 81.9% of the variation. The probabilities of comprehensive knowledge varied by a factor of 1.12 (MOR) in the null model. According to Model I’s intra-class correlation value, 8.8% of the variation in comprehensive knowledge results from individual differences. The likelihood of comprehensive knowledge varied by 1.11 times between low and high comprehensive knowledge in the Model (I). The likelihood of comprehensive knowledge varied by 1.10 times between low and high comprehensive knowledge in Model II. Based on the ICC value from Model II, 8.4% of the variability in comprehensive knowledge was attributed to cluster variations. The likelihood of comprehensive knowledge varied by 1.09 times between low and high comprehensive knowledge in the final (Model III), which attributed roughly 6.1% of the variation in the likelihood of comprehensive knowledge to both individual and community-level variables. The best model, based on deviance, was the one with the lowest deviance (Model III) (Table 2).

## Discussion

This study aimed to assess comprehensive knowledge about HIV and AIDS and associated factors among AGYW in Tanzania using a secondary analysis of a nationwide survey. The observed prevalence of comprehensive HIV and AIDS knowledge among AGYW at 46.2% aligns with findings from several contexts but indicates room for improvement. This prevalence is comparable to the overall regional estimate of 41.6% reported across 30 SSA countries, where comprehensive knowledge ranged widely from as low as 15.0% in Congo to as high as 74.3% in Rwanda (22). Similarly, a study in Malawi reported a slightly lower prevalence of 42.2% among AGYW, highlighting consistent challenges in achieving widespread HIV awareness in the region (23). In contrast, Rwanda reported a higher prevalence of 53.6%, reflecting the impact of intensified HIV educational programs and media outreach (3). Globally, comprehensive knowledge among AGYW remains suboptimal, with less than half of young women demonstrating full understanding of HIV transmission and prevention (3, 22). These variations emphasize the critical role of specific interventions in increasing comprehensive HIV and AIDS knowledge. This prevalence calls for renewed efforts to expand comprehensive sexuality education and accessible information dissemination to close existing knowledge gaps and reduce HIV incidence among this vulnerable population.

The analysis revealed several factors that are correlated with comprehensive HIV and AIDS knowledge. Among these factors is the education level of respondents was noted to influence. The finding showed that adolescent AGYW with primary and secondary education are significantly more likely to be knowledgeable compared to those with no formal education, which is consistent with numerous studies (22). Research has repeatedly shown that educational attainment plays a pivotal role in enhancing knowledge about HIV transmission and prevention (24). Similarly, findings from Malawi highlight that higher educational levels positively influence overall HIV and AIDS knowledge among young women, Collectively, these results underscore the essential role that both primary and secondary education play in empowering AGYW with knowledge necessary to combat the spread of HIV, highlighting the need for policies that promote universal education and integrate comprehensive HIV and AIDS education into school programs.

The result, indicating that AGYW in the richest wealth quintile are more likely to have comprehensive HIV and AIDS knowledge, is consistent with findings from multiple studies. Wealth status has been repeatedly demonstrated as a significant determinant of HIV knowledge, where those from wealthier households generally have better access to education, healthcare information, and media exposure (25). Similarly, studies in Nigeria revealed that women in the highest wealth quintiles are significantly less likely to have low HIV-related knowledge compared to women in poorer quintiles, highlighting the protective effect of economic advantage on HIV awareness (26). The intersection of wealth and education amplifies these disparities because wealthier families are better positioned to support higher educational attainment, which in turn enhances HIV knowledge (3). The findings show the urgent need to bridge the knowledge gap among poorer AGYW by expanding equitable access to health education and HIV prevention resources.

AGYW who have ever tested for HIV exhibit significantly higher odds of possessing comprehensive HIV and AIDS knowledge, consistent with multiple studies that demonstrate the positive relationship between HIV testing and enhanced HIV knowledge (3). Furthermore, meta-analyses related to the DREAMS program have suggested that increased uptake of HIV testing correlates with greater HIV status awareness and knowledge gains (27). This finding showed that HIV testing services serve not only as a gateway to treatment but also as a critical intervention point for enhancing HIV and AIDS knowledge to promote informed and safer sexual behaviors.

The data indicate that at the community level, various geographical zones in Tanzania mainland exhibit significantly higher odds of having comprehensive HIV knowledge when compared to Zanzibar (Tanzania island). These pronounced geographical disparities in HIV knowledge may be explained by several distinguishing factors between Zanzibar and mainland Tanzania regions (28–30). Notably, HIV prevalence itself varies dramatically, with mainland Tanzania showing 4.5 percent prevalence compared to only 0.4 percent in Zanzibar, which may influence the intensity and focus of HIV education programs (8). Zanzibar faces significant healthcare infrastructure challenges, with only 0.4 doctors per 1,000 people compared to a world average of 1.6, potentially limiting access to comprehensive health education (31). Additionally, the regions operate under different educational policy frameworks, with mainland Tanzania following the Education Sector Development Plan (2016/17–2020/21) while Zanzibar implements its separate Zanzibar Education Development Plan II (2017/2018–2021/2022) which initiate early the HIV and AIDS prevention knowledge early (10, 32). These structural differences in healthcare infrastructure, educational systems, and epidemiological contexts across the zones likely contribute to variations in HIV knowledge accessibility and quality (10, 28–30). Addressing these geographical inequalities by tailoring interventions to the specific infrastructural, epidemiological, and educational contexts of lower-performing zones could be vital for improving equitable HIV and AIDS awareness and prevention efforts nationwide.

In response to these knowledge gaps, the Tanzania government has adopted comprehensive policy decisions including the implementation of comprehensive sexuality education into the curriculum, a re-entry program for children who drop out, and amendments to the HIV and AIDS Act to lower the age of consent for HIV testing and allow HIV self-testing (33). Tanzania government has also developed an Integrated Health Sector HIV, Viral Hepatitis and Sexually Transmitted Infections National Strategic Plan to contribute to ending the three epidemics by 2030 (34).

### Strengths and limitations of the study

The strength of this study data from the nationally representative 2022 TDHS, allowing for robust, generalizable findings across various regions and populations of adolescent girls and young women in Tanzania. Utilizing Andersen’s Behavioral Model as a guiding framework offers a comprehensive perspective by integrating predisposing, enabling, and need factors at both individual and community levels, which enhances the depth of analysis regarding determinants of comprehensive HIV and AIDS knowledge. The application of multilevel logistic regression accounts for hierarchical data structures and community-level influences, further strengthening the analytical validity. However, as a cross-sectional study, it is limited in establishing causal relationships among variables. Self-reported data may introduce social desirability and recall biases, potentially affecting the accuracy of responses related to sensitive issues such as sexual behavior and HIV knowledge.

## Conclusion

The study identifies key determinants of comprehensive HIV and AIDS knowledge among Tanzanian AGYW, revealing significant influences from demographic factors such as age and marital status, socio-economic and educational variables, behavioral factors including sexual experience and prior HIV testing, and contextual community-level factors like geographical zones. Andersen’s Behavioral Model effectively captures the multifactorial nature of HIV and AIDS knowledge acquisition, illustrating how predisposing, enabling, and need factors collectively influence awareness. Despite notable progress in HIV education, gaps persist, especially among AGYW, underscoring the need for focused, diversified interventions. By advancing understanding of these determinants, the study provides crucial evidence to inform tailored HIV prevention strategies aimed at enhancing knowledge and reducing infection rates within this vulnerable population and improving safer sex practice.

### Implications for practice, policy reform, and future research

The findings highlight the urgent need to prioritize comprehensive HIV and AIDS education among adolescent girls and young women, particularly targeting younger age groups and those with limited access to media and health services. Practitioners should make perfect use of opportunities during health facility visits and community outreach to reinforce accurate HIV knowledge. Integrating HIV education into school curricula and expanding digital and mass media campaigns can enhance reach and impact. Policymakers should also address structural barriers by improving access to inclusive, youth-friendly services and reducing stigma that hampers information uptake. Future research should employ longitudinal and intervention-based designs to clarify causal relationships and evaluate the effectiveness of specific educational strategies. Additionally, exploring the influence of emerging digital technologies and social determinants in diverse Tanzanian contexts will further strengthen efforts to close HIV knowledge gaps and support the national goal of ending HIV and AIDS as a public health threat by 2030.

## Data Availability

The datasets presented in this study can be found in online repositories. The names of the repository/repositories and accession number(s) can be found at: https://dhsprogram.com/data/dataset_admin/index.cfm.
